# Cognitive trajectories during the menopausal transition

**DOI:** 10.3389/frdem.2023.1098693

**Published:** 2023-01-27

**Authors:** Stephanie Than, Chris Moran, Richard Beare, Amanda Vincent, Emma Lane, Taya Annabelle Collyer, Michele L. Callisaya, Velandai Srikanth

**Affiliations:** ^1^Academic Unit, Peninsula Clinical School, Central Clinical School, Monash University, Melbourne, VIC, Australia; ^2^Department of Geriatric Medicine, Peninsula Health, Melbourne, VIC, Australia; ^3^National Centre for Healthy Ageing, Melbourne, VIC, Australia; ^4^School of Public Health and Preventive Medicine, Monash University, Melbourne, VIC, Australia; ^5^Developmental Imaging, Murdoch Children's Research Institute, Melbourne, VIC, Australia; ^6^Monash Centre for Health Research and Implementation, School of Public Health and Preventative Medicine, Monash University, Melbourne, VIC, Australia; ^7^Department of Endocrinology, Monash Health, Melbourne, VIC, Australia

**Keywords:** sex, dementia, cognition, menopause, hormonal therapy

## Abstract

**Aims:**

Female sex is associated with an increased prevalence of dementia. Menopause may have a role to play in explaining sex differences in cognition, and possibly the risk of future dementia. We aimed to determine if the rate of cognitive decline differed between stages of the menopausal transition.

**Materials and methods:**

Women with data on menopause and longitudinal cognitive function from the UK Biobank study were stratified into three groups: premenopausal, perimenopausal and postmenopausal. We studied associations of these menopause groups with rate of change in reaction time, verbal-numeric reasoning, prospective memory, visual memory and attention/working memory, adjusted for age, education, ethnicity and APOEε4 genotype. We also explored the effect of menopausal hormonal therapy (MHT) use and cross-sectional brain magnetic resonance imaging (MRI) volumes on these models.

**Results:**

We included 15,486 women (baseline mean age 52 years) over a mean duration of 8 years. An interaction between menopausal group status and time was found for reaction time (*p* < 0.01). Compared with premenopausal women, the rate of increase (worsening) in reaction time was least in postmenopausal women (β = −1.07, p for interaction = 0.02). In general, compared with premenopausal women, perimenopausal and postmenopausal women had overall poorer performance in fluid intelligence and memory over the study duration, with no difference in rates of change. The models were unaffected by MHT use and brain volume measures.

**Conclusions:**

Perimenopause and post-menopause are associated with cognitive changes. Psychomotor speed appears to be most sensitive to the menopause transition, whereas other cognitive functions may be less susceptible. More sensitive structural or functional brain imaging may be required to understand the underlying neural basis for these findings.

## 1. Introduction

Female sex is associated with an increased prevalence of dementia, with women comprising two-thirds of affected individuals (Alzheimer's Association, 2019). The reasons underlying this are poorly understood, with increased longevity alone being unlikely to completely account for sex differences in dementia risk. It is likely that subtle changes in cognitive function develop decades prior to the emergence of cognitive impairment. In women, it is important to consider menopause, an important midlife event, as a contributor to subsequent dementia risk (Brinton et al., 2015).

The perimenopausal period, when ovarian sex hormone levels fluctuate and the post-menopausal period, when sex hormone concentrations are markedly reduced (Weber et al., 2014), are frequently associated with a subjective change in cognitive performance, including difficulties with recall, attention and language (Sullivan Mitchell and Fugate Woods, 2001). Although most women appear to transition through perimenopause without long-term adverse effects, a substantial proportion of women emerge with an increased risk of cognitive decline (Brinton et al., 2015). Measuring these cognitive changes and whether changes at different menopausal stages are causally associated with later life dementia remains a topic of ongoing debate.

To date, many studies have focused on single cognitive domains such as verbal memory as the brain areas that serve this function are disproportionately rich in estrogen receptors (McEwen, 2002), with conflicting findings (Meyer et al., 2003; Fuh et al., 2006; Greendale et al., 2009, 2010; Epperson et al., 2013; Weber et al., 2013). Variations in study design and size may have led to these inconsistencies. These studies are also limited by not including the role of brain structure, a marker of dementia risk (Marquis et al., 2002), in explaining any associations they report.

We therefore aimed to study the associations between stages of menopausal transition and cognitive function longitudinally in a large sample of women who participated in the UK Biobank study. We also aimed to explore whether structural brain imaging measures and the use of menopausal hormonal therapy (MHT) mediated any observed associations.

## 2. Materials and methods

### 2.1. Sample description

The data used for this analysis were obtained from the UK Biobank (http://www.ukbiobank.ac.uk/). The UK Biobank was launched as an international open-access resource with the primary aim of improving the prevention, diagnosis and treatment of a wide range of diseases of middle and old age. Between 2006 and 2010, a population cohort of over 500,000 individuals aged 40–69 years who were registered with the National Health Service (NHS) and resided within traveling distance of 22 assessment centers across the UK were recruited (Sudlow et al., 2015). Full details of recruitment and other key procedures with UK Biobank have been published previously and are available on www.ukbiobank.ac.uk. Initial and subsequent assessments collected extensive health and lifestyle information from detailed questionnaires, physical and cognitive measures, biological samples (blood, urine, saliva), and samples for genetic analysis. The methods used to collect data included a series of touchscreen questions, nurse-led interviews and online assessments. From 2014, the UK Biobank began inviting 100,000 of the original participants for brain, heart and abdominal magnetic resonance imaging (MRI). At time of writing, the UK Biobank was still accruing imaging data from the first repeat imaging visit.

Cognitive testing was first administered to the entire UK Biobank cohort at the initial assessment visit, attended by 273,375 women. To be eligible for the current analysis, spanning the first to the fourth follow up-visits, the following were required: (1) menopause status and cognitive performance data collected at ≥2 separate assessment visits; and (2) age between 40 and 60 years of age at the first visit. Given our aim to study natural menopausal transitions during midlife, individuals were excluded if they had a history of a bilateral oophorectomy or breast cancer (as treatments of breast cancer can induce early menopause).

### 2.2. Standard protocol approvals, registrations and patient consents

Written informed consent was obtained from all participants and details are available at http://www.ukbiobank.ac.uk/. The UK Biobank approved the study application (Project ID: 24954) and obtained ethics approval from the UK Biobank Research Ethics Committee (reference 11/NW/0382). We also received ethics approval from the Monash University Human Research Ethics Committee (Project ID: 18734).

### 2.3. Cognitive outcomes

Cognitive tests were administered *via* a touchscreen interface (Lyall et al., 2016). These included measures of reaction time, attention/working memory, verbal-numeric reasoning, visual memory and prospective memory. Two tests were included in the protocol throughout the UK Biobank baseline phase (reaction time and pairs matching), two tests were introduced in the final two years of recruitment (reasoning and prospective memory), and one test (attention/working memory) was introduced in the final 2 years and then subsequently discontinued due to time constraints. The sample size therefore varies across tests.

#### 2.3.1. Reaction time

Participants completed a timed test of symbol matching. The score was the mean response time in milliseconds across trials (data field 20023). A higher score indicates poorer performance in psychomotor speed.

#### 2.3.2. Verbal-numeric reasoning

A fluid intelligence test comprising of verbal and numerical problems was presented and participants were requested to select the correct response from multiple options. The score is a total of correct responses, with a maximum possible score of 13 (data field 20016). A higher score indicates better performance in fluid intelligence.

#### 2.3.3. Prospective memory

Early in the touchscreen cognitive section, participants were advised: “At the end of the games we will show you four colored symbols and ask you to touch the blue square. However, to test your memory, we want you to actually touch the Orange Circle instead” (“Prospective memory result; data field 20018). Later in the assessment, they were presented with the task. Participants were scored dichotomously, depending on whether they completed the task on first attempt or not, with failure reflecting poorer delayed recall.

#### 2.3.4. Visual memory

A *pairs matching test using a* random array of symbol cards was presented on the screen. Participants were asked to memorize the positions of these matching pairs. The cards were then turned face down and participants had to select the matched pairs from memory while making as few errors as possible. There were a 3-pair and 6-pair version for the test; we chose the 6-pair version because there was more scope for score variation. The score was the number of errors that each participant made trying to select the pairs (data field 399). Higher scores reflect poorer performance in visual memory.

#### 2.3.5. Attention/working memory

*A numeric memory test* whereby participants were shown a two-digit number which they were asked to recall after a brief pause. The string of digits presented increased by one until the participant made an error, or they reached the maximum of twelve digits. The score was the maximum number of digits correctly recalled (“Maximum digits remembered correctly”; [data field 4282]). A higher score indicates better performance in attention/working memory.

A detailed description of each of these tests and basic descriptive statistics can be found in the UK Biobank data showcase (https://biobank.ndph.ox.ac.uk/showcase/label.cgi?id=100026).

### 2.4. Menopause status and MHT use

Menopause status at each assessment visit was classified using two separate variables. First, as a self-reported response to the question: “Have you had your menopause (periods stopped)?” Second, participants >60 years of age were deemed to be postmenopause regardless of their survey response (Treloar, 1981). Individuals were excluded if they had characterized themselves as “postmenopausal” at an earlier visit, and then “premenopausal” at a subsequent visit.

Individuals were then placed into the following groups: (1) “premenopausal” (women who reported being premenopausal at all timepoints), (2) “perimenopausal” (women who reported being premenopausal at baseline, and postmenopause in subsequent assessments), and (3) “postmenopausal” (women who reported being postmenopausal at all timepoints).

Use of MHT was determined *via* two methods and combined to form one measure (ever used: yes/no). First, by the response to the questions: “Have you ever used hormone replacement therapy?” (data field 2814) and “Do you regularly take any of the following medications?” (options included MHT) (data field 6153). Second, we classified use of MHT with data obtained through verbal interview by a trained nurse at each visit. If the participant indicated that they were taking regular prescription medication, the interviewer was prompted to record the name of the medication (data field 20003). Using the list employed by clinic nurses to code medical treatments, drugs used for MHT were identified and coded by an endocrinologist (A.J.V.).

### 2.5. Co-variables

Age (years) was calculated as age at attendance to the assessment center. Education attainment was defined as having a tertiary degree or not. Race/ethnicity (white or non-white) was included because there are race-related disparities in the stages of life at which women enter the menopausal transition (Sammel et al., 2009). Participants who answered “prefer not to answer” or “not sure” to touchscreen questions were coded as having missing data (<5%).

### 2.6. Brain MRI volumes

The UK Biobank collected brain imaging data at the third cognitive assessment visit. We included cross-sectional volumetric brain MRI data from this imaging visit alone. The UK Biobank MRI acquisition protocol and pipeline for generation of Imaging Derived Phenotypes (IDPs) have been described previously (Alfaro-Almagro et al., 2018). Brain MRI scans were acquired on a 3T Siemens Skyra using a 32-channel head coil. We used the following IDPs, derived from T1 weighted and T2-weighted fluid attenuated inversion recovery (FLAIR) scans, in our analyses: total brain volume (TBV), total and regional gray matter volume (GMV), white matter volume (WMV), total hippocampal volumes (HV), and white matter hyperintensity volume (WMHV). We used brain volumes normalized for head size. Normalization of brain tissue volumes for head size was carried out using a SIENAX-style analysis (Structural Image Evaluation, using Normalization, of Atrophy: Cross-sectional) (Smith et al., 2002). Where volumes were provided but not normalized, we multiplied raw IDP values by the T1-based “head size scaling factor” provided.

### 2.7. Statistical analyses

Sample characteristics were summarized, stratified by the three menopausal groups. Using a general linear mixed effects model, we fitted growth curves for each participant, each with an intercept (score at cognitive baseline) and a linear aging effect (decline or improvement at constant slope). Key comparisons for this analysis were between menopausal group level effects. Models included random effects for the intercept and slope. The intercepts were allowed to vary by time-invariant demographic characteristics (baseline age, educational level and race/ethnicity). The slopes were allowed to vary by number of years since first assessment. We tested for statistical interactions using product terms between time (years) and menopausal group in predicting cognitive outcomes.

In secondary analyses, we explored the effect of introducing MHT use into the models examining the associations between menopause groups and cognition. To understand the contribution of brain structure to menopause-cognition associations, we compared the mean total brain, gray matter, white matter, hippocampal and white matter hyperintensity volumes between the three menopause groups. We then added each of these brain volume measures to models of the associations between menopause groups and cognition. Given this was an exploratory study, we did not want to limit our ability to detect signals of interest by adjusting *p*-values for multiple comparisons (Althouse, 2016). All statistical analyses were performed using R version 4.0.1.

## 3. Results

### 3.1. Sample characteristics

Baseline characteristics of the participants are summarized in Table 1. The mean age of the participants at the first assessment was 51.6 years, with an age range between 40.1 and 60.0 years. Of the 15,486 individuals included in the study, 1,722 were premenopausal (mean age 43.9 ± 2.6), 5,521 were perimenopausal (mean age 48.1 ± 3.6), and 8,243 were postmenopausal (mean age 55.5 ± 3.2). Participants visited the assessment center up to four times over a mean follow up period of 8.3 years. The prevalence of cardiometabolic risk factors and conditions such as hypertension, hyperlipidemia, ischemic heart disease and stroke was greater in the postmenopausal group compared to premenopausal and perimenopausal groups (all *p* < 0.05). Current MHT use at baseline was most prevalent in postmenopausal (9%), compared to perimenopausal (2%) and premenopausal (0.4%) groups. Proportions of participants carrying at least one apolipoprotein E ε4 allele were similar between groups (~27%) (all *p* > 0.05).

**Table 1:** Baseline sample characteristics.

	**Pre-menopause** **Mean ±SD** **or *n* (%)**	**Perimenopause** **Mean ±SD** **or *n* (%)**	**Post-menopause** **Mean ±SD** **or *n* (%)**
*N*	1,722 (11)	5,521 (36)	8,243 (53)
Age (years)	43.9 ± 2.6	48.1 ± 3.6	55.5 ± 3.2
Tertiary degree	898 (52.1)	2,788 (50.5)	3,844 (46.6)
White race/ethnicity	1,625 (94.5)	5,286 (95.9)	8,003 (97.3)
Hypertension	331 (19.2)	1,135 (20.6)	2,477 (30.0)
Diabetes	37 (2.2)	128 (2.3)	189 (2.3)
Hyperlipidemia	38 (2.2)	167 (3.0)	706 (8.6)
Ischemic heart disease	17 (1.0)	68 (1.2)	236 (2.7)
Stroke	40 (2.3)	150 (2.7)	288 (3.5)
Past or current smoker	524 (30.4)	1,693 (30.7)	3,052 (37.0)
Depression (self-report)	518 (30.8)	1,358 (25.4)	1,857 (23.3)
Waist-hip ratio	0.79 ± 0.1	0.79 ± 0.1	0.80 ± 0.1
Age at menarche (years)	13.0 ± 1.6	13.0 ± 1.6	13.0 ± 1.6
Age at menopause (years)	NA	51.8 ± 3.4	49.7 ± 4.8
Current MHT use	7 (0.4)	114 (2.1)	753 (9.1)
Ever used MHT	127 (7.4)	1,132 (20.5)	3,093 (37.5)
Ever used oral contraceptive pill	1,522 (88.4)	4,964 (89.9)	7,435 (90.2)
Apolipoprotein E ε4 carrier	452 (26.2)	1,570 (28.4)	2,359 (28.6)
Duration of follow up (years)	8.1 ± 1.7	9.3 ± 1.6	8.9 ± 1.7

SD, Standard deviation; MHT, menopausal hormone therapy.

### 3.2. Baseline cognitive performance by menopausal status

Unadjusted menopausal group differences in cognitive test scores at baseline are reported in Table 2. Premenopausal women displayed better cognitive performance than postmenopausal women in all domains (all *p* < 0.05). Perimenopausal women tended to have poorer performance than premenopausal women in all cognitive domains, but these differences were only statistically significant for reaction time, visual memory and attention/working memory. Postmenopausal women had poorer cognitive performance than perimenopausal women in all cognitive domains but these differences were only statistically significant (*p* < 0.05) in the reaction time and visual memory domains.

**Table 2:** Baseline cognitive scores and brain MRI volumes of menopausal groups.

	**Pre-menopause** **Mean ±SD** **or *n* (%)**	**Perimenopause** **Mean ±SD** **or *n* (%)**	**Post-menopause** **Mean ±SD** **or *n* (%)**
**Baseline cognitive test scores (total available)**			
Reaction time* (*n* = 14984)	505 ± 88.8	516 ± 88.0	548 ± 99.8
Verbal-numeric reasoning (*n* = 4,551)	6.81 ± 2.07	6.59 ± 2.02	6.73 ± 2.01
Prospective memory (*n* = 4,617)	579 (33.6%)	1294 (23.4%)	2210 (26.8%)
Visual memory^*^ (*n* = 15,225)	3.17 ± 2.47	3.53 ± 2.75	3.86 ± 3.02
Attention/working memory (*n* = 1,020)	7.06 ± 1.35	6.79 ± 1.23	6.87 ± 1.22
**Baseline brain MRI volumes (total available)**			
Total brain volume (*n* = 11,458)	1,567 ± 60.4	1,546 ± 63.2	1,502 ± 62.5
Gray matter volume (*n* = 11,458)	848 ± 35.8	835 ± 38.4	805 ± 37.5
White matter volume (*n* = 11,458)	719 ± 36.9	711 ± 37.7	697 ± 38.6
Total hippocampal volume (*n* = 11,456)	10.5 ± 1.0	10.4 ± 1.0	10.3 ± 1.0
White matter hyperintensity volume (*n* = 11,092)	1.7 ± 2.5	2.5 ± 3.4	4.2 ± 4.8

^*^Higher score indicates poorer performance; SD, Standard deviation.

### 3.3. Cognitive performance across time by menopausal status

**Figure 1:**
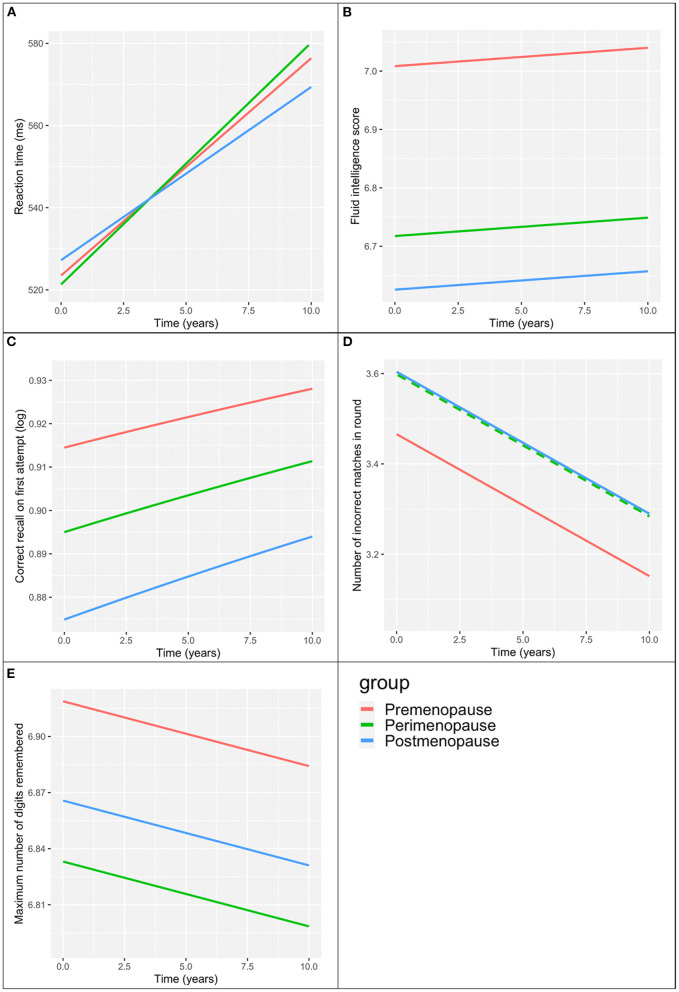
Menopause group associations with cognitive scores over time. **(A)** Reaction time. **(B)** Verbal-numeric reasoning (fluid intelligence score). **(C)** Prospective memory (correct recall on first attempt). **(D)** Visual memory (number of incorrect matches in round). Perimenopause group values (broken green line) the same as the post-menopause group (unbroken blue line). **(E)** Attention/working memory (maximum number of digits remembered). All models adjusted for baseline age, education, ethnicity, time since first visit.

Across time, reaction time slowed in all three groups. We found a statistically significant interaction between menopause status and time (*p* < 0.01). In *post hoc* analysis (Figure 1), we found that the rate of slowing of reaction time was less in the postmenopausal group than the premenopausal group (β = −1.07, *p* = 0.02). Those in the perimenopausal group appeared to have a greater rate of slowing than those in the premenopausal group (β = 0.59) but the difference in slopes did not reach statistical significance (*p* = 0.09).

Table 3 presents the associations between menopausal status and the remaining cognitive tests over time adjusting for baseline age, education and ethnicity. The plots of these associations are presented in Figure 1.

**Table 3:** Associations of menopausal groups with change in cognitive scores over time.

	**Verbal-numeric reasoning** ***n* = 4,551** **β (95% CI),** ***p*-value**	**Prospective memory** ***n* = 4,617** **β (95% CI),** ***p*-value**	Visual memory^*^ n **= 15,225** **β (95% CI),** ***p*-value**	**Attention/working memory** ***n* = 1,020** **β (95% CI),** ***p*-value**
Premenopause	Reference	Reference	Reference	Reference
Perimenopause	−0.29 (−0.45, −0.14), *p* < 0.01	−0.41 (−0.77, −0.06), *p* = 0.02	0.14 (0.02, 0.26), *p* = 0.03	−0.09 (−0.30, 0.13), *p* = 0.44
Post-menopause	−0.38 (−0.59, −0.17), *p* < 0.01	−0.53 (−1.00, −0.07), *p* = 0.02	0.14 (−0.03, 0.31), *p* = 0.11	−0.05 (−0.34, 0.24), *p* = 0.72
Time	0.003 (−0.01, 0.01), *p* = 0.45	1.47 (1.05, 1.90), *p* < 0.01	−0.03 (−0.04, −0.02), *p* < 0.01	−0.003 (−0.01, 0.01), *p* = 0.54

^*^Higher score indicates poorer performance.

Models include baseline age, education and ethnicity.

Of all three groups, postmenopausal women had the poorest verbal-numeric reasoning across time, and premenopausal women had the best performance. The slopes over time were similar across all three groups (interaction term *p*-value >0.2).

Postmenopausal women had the poorest and premenopausal woman demonstrated the best prospective memory across time. There was no interaction between menopausal group and time on prospective memory (*p* > 0.5).

Premenopausal women had the best visual memory of the three groups across time, with perimenopausal and postmenopausal women demonstrating similar performance to each other. There was no interaction between menopausal status and time on visual memory (*p* > 0.1).

Attention/working memory across time was greatest in the premenopausal group and poorest in the perimenopausal group. We did not find an interaction between menopausal status and time on attention/working memory (*p* > 0.6). Coefficients for the co-variables in the above models are presented in Supplementary Tables 1, 2.

### 3.4. Influence of menopause hormone therapy and brain structure

The addition of the MHT use variable did not meaningfully change the associations between menopausal status and cognitive performance reported above. Supplementary Tables 3, 4 present the coefficients of the models including MHT use on cognitive performance.

Brain MRI data was available for 11,458 participants. The mean total brain, gray matter, white matter, hippocampal and white matter hyperintensity volumes for each of the menopausal groups are presented in Table 2. Mean total brain, gray matter, white matter and hippocampal volume were largest in the premenopausal group, and smallest in the postmenopausal group. Mean white matter hyperintensity volume was smallest in the premenopausal group, and greatest in the postmenopausal group.

The addition of brain structure measures did not meaningfully change the association between the interaction of menopausal group with time on reaction time reported above (Supplementary Table 5) or the associations between the menopausal groups and the other cognitive outcomes (Supplementary Table 6).

## 4. Discussion

In this large population-based sample of women at midlife, followed over ~9 years, women transitioning through menopause had poorer cognitive function than premenopausal women in three of five cognitive domains. At baseline, postmenopausal women had poorer cognitive function than premenopausal women in all cognitive domains. The rate of worsening in psychomotor speed was lowest in post-menopause, whereas rates of change in the other cognitive measures were similar. Despite the presence of menopausal group differences in cross-sectional brain volumes, these did not appear to substantially influence the cognitive differences we found. Our results suggest that cognitive changes occur rapidly in the perimenopausal period and that neither menopausal hormone therapy use nor brain volume measures mediate these changes.

The few studies that have examined longitudinal change in cognition during menopause transition consistently report cognitive differences associated with menopause (Meyer et al., 2003; Fuh et al., 2006; Greendale et al., 2009; Epperson et al., 2013). However, there is wide variation in the cognitive domains affected in these studies. Our finding that those transitioning through menopause had poorer cognitive performance in most cognitive domains than those that remained premenopausal may be reflective of the substantially larger sample size in our study.

The absence of a clear worsening of cognition across time in the perimenopausal group suggests that the cognitive changes leading to the differences we report occur rapidly and between the timepoints that cognition was measured. The potential mechanisms underlying these changes are unclear, but the rapidity of the cognitive changes may be reflective of the rapid and pronounced fluctuations of sex hormones that occur during menopause. Studies of other models of acute sex hormone fluctuations such as the luteal phases of the menstrual cycle (Kumar et al., 2013), the onset of puberty (McGivern et al., 2002) and pregnancy (Henry and Sherwin, 2012) have all reported cognitive changes in these states. Our finding that brain structural measures did not mediate the cognitive differences may also be reflective of the rapidity of the change and highlights the need for more sensitive markers of brain health beyond brain structure. For example, previous work has demonstrated that changes in sex hormone levels across the menstrual cycle correlates with brain network efficiency and activity measured using function MRI (Weis et al., 2019).

The contribution of the symptoms associated with sex hormone fluctuations during menopause and cognition are unclear (Maki and Jaff, 2019; Greendale et al., 2020). The menopausal transition is commonly associated with symptoms such as vasomotor symptoms, depression and sleep disturbances (Matthews et al., 1990; Avis et al., 1994; Hardy and Kuh, 2002), which have been shown to be associated with poorer cognition (Maki et al., 2008; Greendale et al., 2010). Such menopausal symptoms become more stable and usually resolve in the postmenopausal setting in parallel with sex hormone concentrations (Harlow et al., 2012). This stability of sex hormone concentration in the postmenopausal setting may explain the slower rate of worsening in psychomotor speed we observe in the postmenopausal group. The results of the Study of Women's Health Across the Nation support this potential explanation, describing impaired learning during menopause transition that returned to premenopausal levels in post-menopause (Greendale et al., 2009).

In our analyses, MHT use and MRI measures of brain structure appeared to have minimal effects on the associations observed. The role of MHT in offsetting symptoms or down-stream effects following menopause, including dementia (Resnick and Henderson, 2002), remains unclear and further research is required (Maki and Jaff, 2022). The absence of a mediating or modifying effect of MRI measures of brain health on the links between menopause status and cognition suggests that the effect size of the menopausal transition may not substantial enough to lead to measurable structural brain changes. It is possible that in aging female brains, menopause sets a cascade in progress that could ultimately lead to demonstrable brain structure changes with the use of more sensitive brain imaging modalities such as functional MRI or measures of connectivity.

Our study has several strengths and limitations. Strengths include our use of a large sample of people with objective and detailed measures of cognition longitudinally and brain structure cross-sectionally. The main limitation of our study related to the categorization of menopausal status. We relied on self-report to assign menopause status. As such, it is possible that women who self-described as pre- or post-menopausal were actually in perimenopause. This misclassification error would lead to an underestimation of differences between perimenopause and the two other groups. We were also limited by the availability of brain MRI measures at a single point in time. Longitudinal MRI data is being made available but would have limited utility in our study due to the long timeframe between scans. A study examining the effects of perimenopause would benefit from more frequent and sensitive cognitive and brain assessments. As this was not a major question informing the development of UK Biobank, this data is not available. Similarly, data relating to menopause-related symptoms (such as alterations in mood and sleep), and duration of MHT use or other medications to manage menopause symptoms were not available. Our study design also prevented us from providing definitive nuanced detail regarding the influence of MHT and other potential confounders such as age. Both age and MHT use were strongly related to menopausal status in our study. Although we attempted to reduce the influence of these factors in our analyses, it remains likely that the relationships between menopause, age and MHT use on cognition are complex and intertwined (Than et al., 2021).

In summary, our findings support the association of menopausal transition with poorer cognition, with this stage of life possibly representing a critical period of cognitive aging for women. Future work should determine the neurobiological basis for this and whether there are long-term impacts on dementia risk.

## Data availability statement

The data analyzed in this study is subject to the following licenses/restrictions: Data available on application to UKBiobank. Requests to access these datasets should be directed to https://www.ukbiobank.ac.uk/enable-your-research/apply-for-access.

## Ethics statement

The studies involving human participants were reviewed and approved by UK Biobank Research Ethics Committee (reference 11/NW/0382). The patients/participants provided their written informed consent to participate in this study.

## Author contributions

ST, CM, RB, MC, AV, and VS contributed to study conception and design. ST, RB, EL, and TC contributed to data cleaning and statistical analyses. ST wrote the first draft of the manuscript. CM and VS provided oversight and guidance of the writing of the manuscript. CM and AV wrote sections of the manuscript. All authors contributed to manuscript revision, read, and approved the submitted version.
