# Supplementary material for: Cognitive trajectories during the menopausal transition
Source: Front Dement. 2023 Jan 27;2:1098693. doi: 10.3389/frdem.2023.1098693 (PMC11285668; doi:10.3389/frdem.2023.1098693)
Supplement: Supplementary file 1 [file Table_1.DOCX]

***Supplementary Material***

# Supplementary Tables

**Supplementary Table 1** Full model for associations of menopausal groups with cognitive scores across time

|  | **Reaction time** | **Verbal-numeric reasoning** | **Prospective memory** | **Visual memory** | **Attention/working memory** |
| --- | --- | --- | --- | --- | --- |
|  | β (95%CI)  p value | β (95%CI)  p value | β (95%CI)  p value | β (95%CI)  p value | β (95%CI)  p value |
| Premenopause | Reference | Reference | Reference | Reference | Reference |
| Perimenopause | See interaction Supplementary Table 2 | -0.29 (-0.45, -0.14) p<0.01 | -0.41 (-0.77, -0.06) p=0.02 | 0.14 (0.02, 0.26)  p=0.03 | -0.09 (-0.30, 0.13) p=0.44 |
| Postmenopause | See interaction Supplementary Table 2 | -0.38 (-0.59, -0.17) p<0.01 | -0.53 (-1.00, -0.07) p=0.02 | 0.14 (-0.03, 0.31)  p=0.11 | -0.05 (-0.34, 0.24) p=0.72 |
| Baseline age  (years) | 3.79 (3.36, 4.22)  p<0.01 | 0.03 (0.01, 0.04)  p<0.01 | 0.02 (-0.01, 0.05)  p=0.28 | 0.04 (0.03, 0.06)  p<0.01 | -0.004 (-0.02, 0.01)  p=0.68 |
| Education  (tertiary degree) | -7.10 (-9.88, -4.32) p<0.01 | 1.14 (1.05, 1.23)  p<0.01 | 0.19 (-0.01, 0.38)  p=0.06 | -0.18 (-0.26, -0.11)  p<0.01 | 0.25 (0.13, 0.37)  p<0.01 |
| Ethnicity  (white reference) | -32.06 (-39.72, -24.40) p<0.01 | 1.64 (1.40, 1.88)  p<0.01 | 1.26 (0.91, 1.62)  p<0.01 | -0.60 (-0.80, -0.41)  p<0.01 | 0.58 (0.24, 0.91)  p<0.01 |
| Time since first visit  (years) | 5.54 (5.29, 5.80)  p<0.01 | 0.003 (-0.01, 0.01)  p=0.45 | 1.47 (1.05, 1.90)  p<0.01 | -0.03 (-0.04, -0.02)  p<0.01 | -0.003 (-0.01, 0.01)  p=0.54 |
| Intercept | 368.48 (348.00, 388.96) p<0.01 | 3.51 (2.82, 4.20)  p<0.01 | 0.30 (-1.08, 1.67)  p=0.67 | 1.95 (1.39, 2.51)  p<0.01 | 6.43 (5.54, 7.32)  p<0.01 |

**Supplementary Table 2** Associations between menopausal group by time interactions and cognitive scores

|  | **Reaction time** | **Verbal-numeric reasoning** | **Prospective memory** | **Visual memory** | **Attention/working memory** |
| --- | --- | --- | --- | --- | --- |
|  | β (95%CI)  p value | β (95%CI)  p value | β (95%CI)  p value | β (95%CI)  p value | β (95%CI)  p value |
| Premenopause | Reference | Reference | Reference | Reference | Reference |
| Perimenopause | 0.59 (-0.08, 1.27) p=0.09 | 0.003 (-0.02, 0.02) p=0.74 | 0.08 (-0.91, 1.08) p=0.87 | -0.01 (-0.04, 0.01) p=0.38 | -0.003 (-0.03, 0.03) p=0.84 |
| Postmenopause | -1.07 (-1.96, -0.19)  p=0.02 | 0.015 (-0.01, 0.04)  p=0.27 | -0.28 (-1.32, 0.75) p=0.59 | -0.02 (-0.06, 0.01) p=0.11 | -0.008 (-0.04, 0.03) p=0.69 |

Model variables include baseline age, education and ethnicity.

**Supplementary Table 3** Associations between menopausal group and cognition across time with additional adjustment for menopausal hormonal treatment.

|  | **Reaction time** | **Verbal-numeric reasoning** | **Prospective memory** | **Visual memory** | **Attention/working memory** |
| --- | --- | --- | --- | --- | --- |
|  | β (95%CI)  p value | β (95%CI)  p value | β (95%CI)  p value | β (95%CI)  p value | β (95%CI)  p value |
| Premenopause | Reference | Reference | Reference | Reference | Reference |
| Perimenopause | See interaction Supplementary Table 4 | -0.27 (-0.43, -0.12) p<0.01 | -0.39 (-0.74, -0.04) p=0.03 | 0.12 (-0.01, 0.24) p=0.06 | -0.07 (-0.29, 0.15) p=0.52 |
| Postmenopause | See interaction Supplementary Table 4 | -0.34 (-0.55, -0.13)  p<0.01 | -0.49 (-0.95, -0.03)  p=0.04 | 0.09 (-0.08, 0.26)  p=0.31 | -0.003 (-0.29, 0.29)  p=0.99 |
| Menopausal hormone therapy  use | -0.93 (-4.17, 2.31) p=0.57 | -0.17 (-0.28, -0.06) p<0.01 | -0.20 (-0.42, 0.01) p=0.06 | 0.19 (0.10, 0.28) p<0.01 | -0.22 (-0.36, -0.08) p<0.01 |

Model variables include baseline age, education and ethnicity

**Supplementary Table 4** Associations between menopausal group by time interactions and cognition across time with additional adjustment for menopausal hormonal treatment

|  | **Reaction time** | **Verbal-numeric reasoning** | **Prospective memory** | **Visual memory** | **Attention/working memory** |
| --- | --- | --- | --- | --- | --- |
|  | β (95% CI)  p value | β (95% CI)  p value | β (95% CI)  p value | β (95% CI)  p value | β (95% CI)  p value |
| Premenopause | Reference | Reference | Reference | Reference | Reference |
| Perimenopause | 0.59 (-0.08, 1.27)  p=0.09 | 0.003 (-0.02, 0.02) p=0.74 | 0.09 (-0.91, 1.09)  p=0.86 | -0.01 (-0.04, 0.01)  p=0.40 | -0.003 (-0.03, 0.03) p=0.84 |
| Postmenopause | -1.08 (-1.96, -0.19)  p=0.02 | 0.014 (-0.01, 0.04)  p=0.28 | -0.28 (-1.31, 0.76) p=0.60 | -0.02 (-0.05, 0.01) p=0.12 | -0.007 (-0.04, 0.03) p=0.71 |
| Menopausal hormone therapy  use | -0.96 (-4.20, 2.28) p=0.56 | -0.17 (-0.28, -0.06) p<0.01 | -0.20 (-0.42, 0.02) p=0.07 | 0.19 (0.10, 0.28) p<0.01 | -0.22 (-0.36, -0.08)  p<0.01 |

Model variables include baseline age, education and ethnicity

**Supplementary Table 5** Associations between menopausal group by time interactions and reaction time adjusting for various brain structure volumes

|  | **Reaction time** |
| --- | --- |
| **Covariable (Volume)** | β (95% CI)  p value |
| **Total brain** |  |
| Premenopause | Reference |
| Perimenopause | -0.09 (-0.85, 0.68)  p=0.83 |
| Postmenopause | -1.20 (-2.21, -0.19)  p=0.02 |
| **Gray matter** |  |
| Premenopause | Reference |
| Perimenopause | -0.09 (-0.85, 0.67)  p=0.82 |
| Postmenopause | -1.22 (-2.23, -0.21)  p=0.02 |
| **White matter** |  |
| Premenopause | Reference |
| Perimenopause | -0.08 (-0.84, 0.68)  p=0.83 |
| Postmenopause | -1.18 (-2.19, -0.17)  p=0.02 |
| **Hippocampal** |  |
| Premenopause | Reference |
| Perimenopause | -0.09 (-8.57, 4.44)  p=0.81 |
| Postmenopause | -1.22 (-4.23, 12.68)  p=0.02 |
| **White matter hyperintensity** |  |
| Premenopause | Reference |
| Perimenopause | -0.07 (-0.85, 0.71)  p=0.86 |
| Postmenopause | -1.15 (-2.18, -0.11)  p=0.03 |

Model variables include baseline age, education and ethnicity

**Supplementary Table 6** Associations between menopausal group and cognition across time with additional adjustment for brain structure volumes

|  | **Verbal-numeric reasoning** | **Prospective memory** | **Visual memory** | **Attention/working memory** |
| --- | --- | --- | --- | --- |
| **Covariable (volume)** | β (95%CI)  p value | β (95%CI)  p value | β (95%CI)  p value | β (95%CI)  p value |
| **Total brain** |  |  |  |  |
| Premenopause | Reference | Reference | Reference | Reference |
| Perimenopause | -0.38 (-0.55, -0.20) p<0.01 | -0.29 (-0.57, -0.01) p=0.04 | 0.19 (0.05, 0.33) p=0.01 | -0.10 (-0.33, 0.12) p=0.37 |
| Postmenopause | -0.47 (-0.71, -0.23) p<0.01 | -0.50 (-0.88, -0.13) p=0.01 | 0.22 (0.02, 0.41) p=0.03 | -0.02 (-0.32, 0.28) p=0.88 |
| **Gray matter** |  |  |  |  |
| Premenopause | Reference | Reference | Reference | Reference |
| Perimenopause | -0.37 (-0.55, -0.20)  p<0.01 | -0.29 (-0.57, -0.01)  p=0.04 | 0.19 (0.05, 0.34)  p=0.01 | -0.10 (-0.32, 0.13)  p=0.41 |
| Postmenopause | -0.47 (-0.71, -0.23)  p<0.01 | -0.51 (-0.89, -0.13)  p=0.01 | 0.22 (0.02, 0.42)  p=0.03 | -0.01 (-0.32, 0.29)  p=0.92 |
| **White matter** |  |  |  |  |
| Premenopause | Reference | Reference | Reference | Reference |
| Perimenopause | -0.38 (-0.55, -0.20)  p<0.01 | -0.28 (-0.57, -0.004)  p=0.04 | 0.19 (0.05, 0.33)  p=0.01 | -0.11 (-0.33, 0.12)  p=0.35 |
| Postmenopause | -0.46 (-0.70, -0.22)  p<0.01 | -0.50 (-0.88, -0.13)  p=0.01 | 0.22 (0.02, 0.42)  p=0.03 | -0.03 (-0.33, 0.27)  p=0.85 |
| **Hippocampal** |  |  |  |  |
| Premenopause | Reference | Reference | Reference | Reference |
| Perimenopause | -0.37 (-0.55, -0.20) p<0.01 | -0.29 (-0.57, -0.004)  p=0.04 | 0.19 (0.04, 0.33) p=0.01 | 0.00 (-0.03, 0.03) p=0.81 |
| Postmenopause | -0.46 (-0.70, -0.22) p<0.01 | -0.51 (-0.89, -0.13) p=0.01 | 0.22 (0.02, 0.41) p=0.03 | -0.01 (-0.05, 0.03) p=0.62 |
| **White matter hyperintensity** |  |  |  |  |
| Premenopause | Reference | Reference | Reference | Reference |
| Perimenopause | -0.34 (-0.52, -0.16) p<0.01 | -0.26 (-0.55, 0.02) p=0.07 | 0.19 (0.04, 0.34) p=0.01 | -0.11 (-0.34, 0.12) p=0.34 |
| Postmenopause | -0.42 (-0.66, -0.17) p<0.01 | -0.47 (-0.85, -0.09) p=0.02 | 0.20 (0.0003, 0.41) p<0.05 | -0.03 (-0.33, 0.28) p=0.86 |

Model variables include baseline age, education and ethnicity
